# Gender Differences in Self-Estimated Intelligence: Exploring the Male Hubris, Female Humility Problem

**DOI:** 10.3389/fpsyg.2022.812483

**Published:** 2022-02-07

**Authors:** David Reilly, David L. Neumann, Glenda Andrews

**Affiliations:** ^1^School of Applied Psychology, Griffith University, Southport, QLD, Australia; ^2^Menzies Health Institute Queensland, Southport, QLD, Australia

**Keywords:** gender differences, self-estimated intelligence, self-esteem, sex-roles, sex differences, human intelligence, education

## Abstract

Despite evidence from cognitive psychology that men and women are equal in measured intelligence, gender differences in self-estimated intelligence (SEI) are widely reported with males providing systematically higher estimates than females. This has been termed the *male hubris, female humility* effect. The present study explored personality factors that might explain this. Participants (*N* = 228; 103 male, 125 female) provided self-estimates of their general IQ and for Gardner’s multiple intelligences, before completing the Cattell Culture Fair IQ test as an objective measure of intelligence. They also completed the Bem Sex Role Inventory (BSRI) as a measure of sex-role identification, and measures of general and academic self-esteem. Both gender and sex-role differences were observed for SEI, with males and participants of both genders who scored high in masculinity offering higher self-estimates. By comparing estimated and observed IQ, we were able to rule out gender differences in overall accuracy but observed a pattern of systematic underestimation in females. An hierarchical multiple regression showed significant independent effects of gender, masculinity, and self-esteem. Mixed evidence was observed for gender differences in the estimation of multiple intelligences, though moderately sized sex-role differences were observed. The results offer a far more nuanced explanation for the male hubris, female humility effect that includes the contribution of sex role identification to individual and group differences.

## Introduction

“Such is the nature of men, that howsoever they may acknowledge many others to be more witty, or more eloquent, or more learned; yet they hardly believe there be many so wise as themselves.”—Thomas Hobbes, English philosopher.

Intellectual self-image can be a powerful predictor of eventual educational achievement. How we see ourselves intellectually—either as smart, academically capable or possessing more mediocre abilities—can have a profound impact on academic engagement and motivation, the pursuit of intellectual endeavors, persistence in the face of adversity, and self-efficacy beliefs, and even performance on tests of intellectual ability. Psychologists and educators have known this for decades, ever since Rosenthal and Jacobson’s (1968) classic *Pygmalion in the Classroom* study. In this study, the experimenters had students complete a bogus “Harvard intellectual assessment,” and then teacher expectations of individual students were experimentally manipulated by randomly assigning children to be labeled as either “gifted,” ordinary, or below average. Longitudinal testing found that those in the experimental group exhibited significant growth relative to their peers in psychometrically measured IQ one year later. Such an example highlights not only the self-fulfilling prophecy of intellectual self-image (Greven et al., 2009), but also that it can be manipulated and shaped by environmental factors outside of our own control or even awareness. Though the study has at times been criticized on methodological grounds (c.f., Rosenthal, 1995; Snow, 1995), it spurred research into the benefits of teaching a “growth mindset,” and that intelligence is malleable rather than being innately fixed at birth (Dweck, 2016; Yeager et al., 2019).

This brings us to a quite curious phenomenon frequently observed in psychological studies over the last few decades: that, when asked to provide an estimate of their intelligence, males frequently provide higher estimates than females. Indeed, this pattern of gender differences in self-estimated intelligence (SEI) is so universally found across different samples, ages, ethnicities and cultures that it has been termed the *male hubris, female humility* (MHFH) problem by Furnham et al. (2001). It remains so interesting because there is overwhelming consensus in cognitive psychology that males and females *do not differ* in general intelligence; gender differences are only found for specific cognitive abilities like verbal/visual-spatial tasks rather than psychometric intelligence (for a thorough review see Halpern et al., 2011). However our appraisals of our intellect contribute greatly to academic motivation (Dweck, 2002)—students who feel that they are less intellectually capable than their peers are less motivated (Kornilova, 2009). This is particularly so in stereotypically female underrepresented fields like science, technology, engineering and mathematics (Reilly and Hurem, in press). Intellectual self-image also guides course selection (such as the decision to pursue more advanced coursework in high school and college), as outlined by Eccles (2013) expectancy-value theory of achievement motivation: students select coursework that they expect they can reasonably master, and shy away from more challenging subjects if they believe they are not smart enough.

The male hubris/female humility effect has sparked much research across more than thirty countries (Freund and Kasten, 2012; Furnham, 2017). An earlier meta-analysis of studies by Syzmanowicz and Furnham (2011) found the effect to be robust with an average effect size of *d* = 0.37, which is a small to moderate effect size. So, if males and females do not differ in general intelligence but males provide higher estimates of their own intellectual prowess than females, what factors explain this discrepancy?

## Accuracy of Self-Estimated Intelligence

Psychological research has investigated whether people are accurate judges of their intellectual ability generally (irrespective of gender). This arises from several strands of investigation: firstly, whether people are generally sound judges of their intellectual strengths and weaknesses, and secondly, whether there are cognitive biases that affect such evaluations. Furnham (2017) noted that an unresolved research question is whether males over-estimate their actual IQ, females under-estimate IQ, or indeed both, but writes “there are not enough good studies with both self-estimated and test-derived IQ to settle the argument,” p. 110. This may be due to the relative ease with which self-estimates of intelligence may be obtained, but the greater difficulty, time, and expense needed to administer psychometrically valid intelligence tests. There are some examples where a proxy is used, such as a vocabulary test, to investigate the association between self-estimated IQ and intellect (*r* = 0.25, McCrae and Costa, 1985), while others choose to use a test of non-verbal reasoning like the Raven’s Progressive Matrices (*r* = 0.29, von Stumm, 2014). In a review of studies comparing self-estimated and psychometrically assessed intelligence, Paulhus et al. (1998) note that in student subject pool samples, correlations rarely exceed *r* = 0.30 which is a moderately sized effect. They further note that somewhat larger correlations are found in studies that sample from the general population. To provide a frame of reference for evaluating this, self-reports of intelligence have roughly the same predictive validity and accuracy as the situational judgment tests (SJTs) that are widely employed in organizational psychology for predicting cognitive performance (*r* = 0.29 in a meta-analysis by McDaniel et al., 2007). People’s impressions of their intellect are therefore grounded firmly in reality, but their accuracy is subject to distortion by cognitive biases.

### Cognitive Biases

One such bias noted in the literature is the “above-average effect” (Alicke, 1985; Dunning et al., 1989; Kruger, 1999; Kruger and Dunning, 1999), which holds that for socially desirable traits such as competence and intellectual ability, there is a tendency for most people to see themselves as better than the average person. The implication of this, Kruger and Dunning (1999) argue, is that such overly favorable views of their abilities mean that a large proportion of the population is “unskilled and unaware of it,” p. 1121. Such a claim stands in contrast to evidence on the general accuracy of self-estimates of intelligence reviewed above, though the number of studies empirically testing this with psychometrically valid IQ tests are few.

Another bias is the self-esteem bias (Felson, 1981), which is the tendency for people to evaluate themselves in a way that is consistent with their general self-esteem; someone who is high in self-esteem will tend to see themselves as brighter and more capable than someone lacking in self-esteem. While self-esteem is a normally distributed trait, there are frequently observed variations for different subgroups. Gender differences in general and academic self-esteem are well documented (Eccles et al., 1993; Gentile et al., 2009), with boys and men reporting higher general and academic self-esteem than girls and women. Syzmanowicz and Furnham (2011) raised this issue in their meta-analytic review as one possible explanation for the MHFH effect. However, they reported no correlation between self-estimated intelligence and self-esteem, and it seems few studies have actually pursued this line of reasoning (Mirjalili et al., 2011).

### Parental Beliefs, and Socio-Cultural Transmission of Gender Stereotypes

Environmental factors are also likely to contribute to a gender bias in self-estimated intelligence which may be an extension of existing socio-cultural gender stereotypes. Social motives (e.g., boastful pride for males or modesty for females) might explain self-estimates of intelligence. If so, when asked to estimate of *other people’s* intelligence the MHFH effect should not still be present. In the original study by Hogan (1978) into self-estimates of intelligence, participants were also asked to provide an estimate of the intelligence of their mothers and fathers. Fathers were rated as more intelligent than mothers (Hogan, 1978), even though there were no gender differences in general intelligence in the community. The effect has been replicated numerous times (Beloff, 1992; Furnham and Rawles, 1995), but should be interpreted cautiously as it might reflect the systemic educational and occupational inequalities of the time (i.e., higher male educational advancement) rather than genuinely held beliefs that men are inherently “smarter.”

Furnham and Gasson (1998) took a different approach, and instead asked parents to provide an estimation of the intelligence of their own children. Sons were rated as more intelligent than daughters (*d* = 0.67), and this effect has been replicated (Beloff, 1992; Furnham, 2000; Furnham et al., 2002a). Such a pattern of results suggests that environmental factors like gender stereotypes might contribute to the MHFH problem, rather than differential social desirability for intelligence between men and women. Parental beliefs may be a particularly important mechanism in the socialization of gender stereotypes, as parental educational expectations may influence a child’s view of their own capabilities (Frome and Eccles, 1998; Jodl et al., 2001). Parental beliefs and expectations may inadvertently enhance or stifle a developing child’s intellectual self-concept and self-efficacy beliefs: raising a child that feels either bright and capable even in the face of challenges (mastery orientation) or overwhelmed and incapable of more advanced intellectual achievement (learned helplessness). Numerous studies have demonstrated that parental beliefs about their children’s intellectual abilities predict later educational achievement in adolescence and young adulthood (Jodl et al., 2001; Phillipson and Phillipson, 2007; Gunderson et al., 2012; Pinquart and Ebeling, 2019). This may be partly through direct transmission of parental beliefs and expectations, but also because parents can provide or withhold enriching cognitive experiences which can accelerate intellectual development outside of school.

Parents are but one element in a larger ecological system that contributes to intellectual development and intellectual self-image. This system includes the role of teachers and educators in shaping the intellectual self-image of children in their care (Jussim and Harber, 2005; Kollmayer et al., 2018), as well as differential treatment of boys and girls (particularly in gender-typed courses such as mathematics and science). Children’s intellectual self-image is also shaped by media and popular culture (Solbes-Canales et al., 2020), which also plays a part in transmission of cultural gender stereotypes about intellectuality (Nosek et al., 2002; Storage et al., 2020).

### Sex-Role Identification and Self-Estimated Intelligence

Another potential explanation for the MHFH effect may be the contribution of gendered personality traits, and sex-role identification. Bem (1981b) proposed *gender schema theory* as a cognitive account for the way that cultural prescriptions about masculinity and femininity become integrated into our self-concepts. These self-concepts forms internalized standards for regulating our own behavior, and also evaluating that of others through the lens of a gender schema. Now, while boys and girls typically differ in their early socialization experiences (Eccles et al., 1990; Lytton and Romney, 1991), there is also considerable individual variation in the degree to which one acquires stereotypically masculine and feminine personality traits, behaviors and interests- a process termed sex-typing (Kagan, 1964; Kohlberg and Ullian, 1974). The internalized gender schema of each individual differs and is the product of both biological and environmental factors that contribute to their sex-role identity (Tenenbaum and Leaper, 2002; Hines, 2011, 2015; Svedholm-Häkkinen et al., 2018). Highly sex-typed persons are motivated to keep their behavior and self-concept consistent with traditional gender norms of their biological sex (Maccoby, 1990; Martin and Ruble, 2004), and so implicit beliefs about gender and intellectuality could translate to higher estimates of intelligence by males and lower estimates by females. For many people their sex-role identification is veridical with their biological sex, but others are more flexible and incorporate a healthy blend of both masculine and feminine personality traits into their self-schema. Researchers have termed this psychological androgyny (Bem, 1984; Spence, 1984; Reilly, 2019), and it has been associated with greater psychological adaptability and less rigid gender schemas. Might sex-role identification act as a better predictor of self-estimated intelligence than the social category of gender?

There are several lines of reasoning that would support such an association. Firstly, as outlined above, it has been hypothesized that self-esteem makes a strong contribution to self-estimated intelligence. While gender differences in self-esteem are frequently reported (Gentile et al., 2009), numerous studies have documented a positive association between masculinity and self-esteem in both men and women (Whitley, 1983; Burnett et al., 1995). This, in turn, might drive higher self-estimates of intelligence. Secondly, there are links between sex-role identification and the development of cognitive ability. Nash’s (1979) sex-role mediation hypothesis proposed that both masculine and feminine sex-roles contribute to cognitive development: masculinity predicts visual-spatial performance (Reilly and Neumann, 2013), while femininity predicts verbal and language abilities (Pajares and Valiante, 2001; McGeown et al., 2011; Reilly et al., 2016). Those higher in masculine and feminine traits may rate their abilities in those domains as higher, which may contribute to their overall impression of intellectuality. Beyer and Bowden (1997) reported the tendency for women to underestimate their performance on stereotypically masculine tasks, but that this underestimation was not found for neutral or feminine tasks. Thirdly, for those with rigid gender schema, male boastfulness and female humility may temper their self-reports and over time shape their self-concept to reflecting implicit gender stereotypes.

Several studies have tested the contribution of sex-role identification to the MHFH effect. The first by Furnham et al. (1999) recruited a small number of subject pool participants, and had them complete the Personal Attributes Questionnaire (PAQ; Spence et al., 1974) which attempts to measure masculinity and femininity as personality traits. Results were inconclusive, though the study was underpowered. A second study by Rammstedt and Rammsayer (2002) recruited a larger sample size and instead used the Bem Sex-Role Inventory (BSRI; Bem, 1981a) which has greater psychometric validity (Choi et al., 2009). Subjects were asked both about their overall intelligence, as well as domain-specific multiple intelligences in line with Gardner’s (1999) typologies. The authors found tentative support for sex-role effects in males, with those scoring higher in masculinity rated their mathematical-logical and general reasoning higher than lower-masculinity peers. However, the authors did not find sex-role effects for the females in their sample. Finally, a study by Storek and Furnham (2012) that recruited intellectually gifted MENSA members found a positive association between masculinity and self-estimated intelligence in both men and women. However, generalizability from such a highly-select sample is questionable. Furthermore, none of these studies included an actual measure psychometric IQ or of self-esteem to determine what role (if any) this played in the MHFH effect.

## General Intelligence Versus Multiple Intelligences

Experts on human intelligence have different views on the nature and structure of intelligence to those of the everyday man and woman. Intelligence is not a unitary construct (Neisser, 1979; Halpern, 2011), and comprises a large number of distinct abilities such as verbal intelligence, mathematical/logical intelligence, emotional intelligence, and so on. Sternberg et al. (1981) examined how lay conceptions of intelligence cluster around a different set of abilities to that of intelligence experts. Sternberg (2000, p. 3) argued that understanding these implicit or lay theories of intelligence was crucial, as “implicit theories of intelligence drive the way in which people perceive and evaluate their own intelligence and that of others.” In reference to the present topic, while gender differences in overall SEI are widely documented, we might see different estimation patterns for certain abilities, such as those stereotypically regarded as masculine or male-dominated (mathematical/analytical, spatial), and those more readily associated with femininity or that are regarded as stronger in females (e.g., verbal and emotional intelligence).

One taxonomy for considering intelligence is Gardner’s (1983, 1999) theory of multiple intelligences. Furnham (2000, 2001) first investigated whether the MHFH effect extended to Gardner’s multiple intelligences, which has since been expanded to encompass seven to nine distinct clusters of abilities depending on the definitions used (Furnham et al., 2001, 2002a,b). Even though intelligence researchers may disagree on the psychometric validity of Gardner’s multiple intelligences, student perceptions of them are important as they may guide course selection. Subjects are typically presented with a definition of each of Gardner’s multiple intelligences, and asked to estimate their intelligence relative to others. These domains are: verbal or linguistic intelligence, logical or mathematical intelligence, spatial intelligence, musical intelligence, bodily-kinesthetic intelligence, interpersonal intelligence, intrapersonal intelligence, naturalistic intelligence, and existential/spiritual intelligence.

Research on self-estimations has revealed a complex and nuanced pattern: while gender differences were almost always found for estimates of general intelligence, they were not reliably found for all of Gardner’s multiple intelligences. Moreover, cross-cultural differences are present. For example, Yuen and Furnham (2006) found that students in Hong Kong did not exhibit significant gender differences for verbal or interpersonal intelligence (stereotypically feminine) but did for all the remaining abilities. However, Furnham et al. (1999, Study 2) found significant gender differences with an English sample for only three of Gardner’s domains: mathematical/logical, spatial and musical intelligence. A review by Furnham (2001) on several of Furnham and colleagues’ studies noted that consistent gender differences were primarily found on stereotypically masculine intellectual abilities (mathematical/logical, and spatial), which Storek and Furnham (2012, 2014) subsequently referred to as domain-masculine intelligence (DMIQ). Furthermore, Storek and Furnham (2013) also found a moderately sized correlation between masculinity and self-estimates for DMIQ, *r* = 0.26, suggesting that there may be sex-role contributions to the effect.

When there are inconsistencies across studies and types of samples, the technique of meta-analysis provides a greater degree of confidence of the robustness of an effect than any single study alone. Syzmanowicz and Furnham (2011) conducted a meta-analysis on self-estimates of general intelligence and for three multiple intelligence domains, reporting moderately large gender differences favoring males for general intelligence, *d* = 0.37, mathematical/logical intelligence, *d* = 0.44, spatial intelligence, *d* = 0.43, and a much smaller difference for verbal intelligence, *d* = 0.07. However, none of the other forms of multiple intelligences were investigated. Moreover, further research is required to determine the extent of gender differences for other domains and to test potential moderators for the self-estimation effects.

## The Present Study

Given the limitations outlined above with previous studies, we set out to explore potential factors that might explain the male-hubris, female humility (MHFH) effect. As Furnham (2017) remarked, there is a paucity of studies comparing self-estimates to psychometrically valid IQ scores, and there are fewer still that include a measure of general self-esteem. Including a measure of sex-role identification would allow us to determine whether social category (male or female) or personality traits (masculinity/femininity) is a better predictor of SEI. Our investigation was primarily exploratory in nature rather than advocating for a particular theory, and this required us to perform additional statistical tests (e.g., that there might be gender differences in psychometric intelligence for our sample due to selection bias) in order to rule them out as alternate explanations. Also, though not the primary focus of the study, previous literature had identified associations between sex-role identification and self-esteem, as well as consistent gender differences. On this basis, it was reasoned that self-esteem might partly explain gender and sex-role differences in self-estimated intelligence (SEI).

The following hypotheses were made:

H1) Males will report higher SEI scores than females for general intelligence.
H2) High masculinity participants (i.e., masculine and androgynous groups) will report higher SEI scores than low masculinity ones (i.e., feminine and undifferentiated), regardless of gender.
H3) Males and high masculinity groups will report higher general self-esteem and academic self-esteem than females and low masculinity groups.
H4) There would be a significant positive correlation between SEI and psychometric intelligence, consistent with past studies (Paulhus et al., 1998).
H5) It is hypothesized that gender, masculinity, and general self-esteem will be associated with SEI, even after controlling for psychometrically measured intelligence.
H6) Masculinity scores would act as a statistical mediator of the relationship between gender and SEI scores.
H7) Gender and sex-role differences will also be found in self-estimates of multiple intelligences, following a similar pattern as observed with general intelligence.

## Materials and Methods

### Participants

Two hundred and twenty-eight participants (103 male, 125 female) with a mean age of 22.62 (SD = 6.30, range = 18–47 years) were recruited from a university subject-pool of students completing a first-year research methods and statistics course. While the majority of these students were completing an undergraduate psychological science degree (53.7%), a large proportion were enrolled in exercise science or physiotherapy (30.7%), followed by health or biomedical sciences (7.4%) and occupational therapy (4%). Only 3% were studying another type of degree. This subject pool was chosen because it included psychology and non-psychology students in order to draw from a broader pool of sex-role categories. Most students were in their first trimester of university and participated prior to receiving their course grade. All participants provided informed consent to a research protocol approved by the Griffith University Human Research Ethics Committee (HREC).

### Procedure

Participants were informed that they were participating in a study on the measurement of human intelligence, and the accuracy of self-estimates. They were provided with a booklet containing the self-estimated intelligence (SEI) measures, followed by the Cattell Cultural Fair IQ Test (CCFIT). Rest periods were provided between each subtest of the CCFIT test to minimize fatigue effects. Following test administration, participants completed surveys measuring self-esteem, sex-role identification, and general demographic information. The surveys were administered after the self-estimated intelligence survey and CCFIT, in order to minimize gender priming effects on SEI and test performance. Participants were tested in small groups (maximum three participants per session) so that compliance with instructions could be monitored and that survey items were read and considered before answering.

### Measures

#### Self-Estimated Intelligence

**FIGURE 1:**
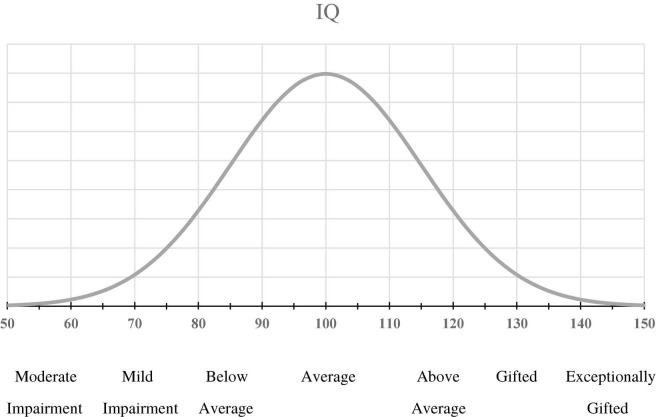
Stimulus material used for self-estimation of intelligence.

Following the methodology of Furnham and Rawles (1995), participants were provided with a simple one page sheet from the booklet which explained in a brief paragraph that the distribution of intelligence in the general population followed a bell curve (see Figure 1 for stimuli) that is normally distributed, with the average IQ score being 100 with a standard deviation of 15. The text of the paragraph was also read aloud by the experimenter to ensure that written instructions were followed. While the properties of the normal distribution were familiar to students in the statistics course, labeled framing anchors were also provided to aid in estimation. Participants were asked to use this scale to provide an estimate of their intelligence relative to other people, and to write this as a whole number.

### Moderate Mild Below Average Above Gifted Exceptionally

#### Impairment Average Gifted

On a subsequent page of the booklet, participants read several paragraphs describing the research of Gardner’s (1999) theory of multiple intelligences, which defined intelligence more broadly than would be typically assessed by an IQ test. Gardner subsequently revised his model of multiple intelligences to include a total of nine separate skills (Verbal and linguistic intelligence, Logical-mathematical intelligence, Spatial, Musical, Bodily-kinesthetic, Interpersonal, Intrapersonal, Naturalistic, and Existential/Spiritual Intelligence). Each skill was accompanied by a brief paragraph description that had been pilot tested for readability. An issue identified in pilot testing was that some participants completed the task extremely quickly with minimal variation in scores across domains. So that participants gave considered and deliberated responses, they were instructed to complete the task one definition at a time, and to record a response *only* after the experimenter had read the paragraph aloud (on the pretense “some participants might come from a non-English background or have reading impairments such as dyslexia, and we want to make sure instructions are clearly understood”). This also ensured that participants had received the appropriate definition for each task, even if they elected not to read the presented material. The definition of existential/spiritual intelligence was phrased for inclusiveness so that it was clear to subjects that this may include but does not require religious practice. Participants responded by providing a numerical IQ score in the same format as for general intelligence.

#### Cattell Culture Fair Test of Intelligence (Cattell et al., 1973)

The CCFIT is a non-verbal measure of fluid intelligence (*gF*), designed specifically to be as free of culture and educational experiences as possible. Additionally, prior research confirmed no gender bias in the CCFIT with equivalent scores for males and females among adult high school graduates (Colom and García-López, 2002). The specific instrument employed was CCFIT Scale 3, Form A intended for use with adult participants. The CCFIT assessment requires inductive reasoning about perceptual patterns, and is comprised of four subtests (series completion, classification, matrices, conditions/typology). Each subtest is completed under strict timing conditions, with items of increasing level of difficulty such that less than 10% of subjects completed all items in the current sample. Although there was no penalty for guessing, two of the subtests require multiple correct responses for the item to be scored correctly. Individual responses were recorded on response sheets that were transcribed and then computer scored for accuracy of scoring. Reliability of the instrument for the current sample was high across the four subtests (Cronbach’s α = 0.72).

The instrument also provides appropriate norms tables to allow for conversion between raw scores and their equivalent IQ (centered around a mean of 100 with a standard deviation of 15), for direct comparability to SEI scores provided by participants. The CCFIT also shows strong convergent validity other tests of general intelligence such as the WAIS with *r* = 0.72 (Cattell et al., 1973), and loads highly against more recently revised intelligence scales (Carroll, 1993).

#### General Self-Esteem

Participants completed the Rosenberg (1965) General Self Esteem Scale, a brief 10 item rating scale that is widely used and demonstrates good psychometric reliability and validity (Sinclair et al., 2010). Participants recorded a response on a 4-point Likert-type scale (ranging from 1 = *“Strongly Agree,”* to 4 = *“Strongly Disagree”*). Sample items include “On the whole, I am satisfied with myself” and “All in all, I am inclined to feel that I am a failure,” with several items being reverse coded (Cronbach’s α = 0.89 for sample).

#### Academic Self-Esteem

There were two measures. Subjects completed a seven-item Academic Self-Esteem scale adapted for this present study from Johnson et al.’s (1983) Academic Self-Esteem subscale, and Bachman’s (1970) Self-Concept of Ability Scale (SCAS). For comparability, subjects endorsed items on the same 4-point scale used for the Rosenberg GSES. Sample items include “I feel confident in my ability to complete university,” and “I am not doing as well at university as I would like to” with negatively worded items that were reverse coded. Subjects also completed the single item Rosenberg Academic Self-Esteem scale, which asks “How do you rate yourself in academic ability compared with those studying your degree” on a 4-point scale. The final response variable incorporated both measures of academic self-esteem, with high reliability (Cronbach’s α = 0.87).

#### Bem Sex-Role Inventory

The 30-item short form of the Bem Sex Role Inventory (BSRI; Bem, 1974, 1981a) was used as a measure of sex-role identification that construes masculinity and femininity as independent constructs on a continuous scale (Reilly, 2019). The BSRI includes 10 masculine, 10 feminine as well as 10 neutral and filler items so that the gendered nature of the instrument is not transparent. Traits are rated on a 7-point Likert scale (from “1 = Never or almost never true of me” to a midpoint of “4 = Occasionally true” and ending in “7 = Always or almost always true of me”). Separate masculinity and femininity scores were produced by averaging responses across each scale, resulting in a continuous score. Participants were also categorized on the basis of a median split of their masculinity (*Mdn* = 4.60) and femininity (*Mdn* = 5.30) scores, to one of four sex-role categories: masculine, feminine, androgynous (high masculinity and high femininity) and undifferentiated (low in both masculine and feminine personality traits). Internal consistency, as assessed by Cronbach’s α, was high in the present sample (masculinity scale α = 0.81, femininity scale α = 0.85) and despite the passage of time since its inception the BSRI remains a valid measure of sex-role identification in modern samples (Choi et al., 2009). For a further review on the psychometric properties of sex-role measures, and why the BSRI remains valid today see Wood and Eagly (2015), and Eagly and Sczesny (2019).

## Results

We present first the sex-role classification for our sample, measured psychometric intelligence, self-estimated intelligence, general and academic self-esteem, followed by hypotheses testing.

### Sex-Role Classification

The distribution of sex-role categories for participants appear in Table 1. As has been found in previous studies, the distribution of sex-role identification is not even in college-aged samples (e.g., feminine-scoring males and masculine-scoring females are underrepresented; Bem, 1981b). Also in line with past studies, independent samples *t-*tests showed that males were significantly higher in BSRI masculinity scores than females, *t*(225) = 3.04, *p* = 0.003, *d* = 0.41, and that females were significantly higher than males in BSRI femininity, *t*(225) = −2.48, *p* = 0.014, *d* = −0.33.

**TABLE 1:** Distribution of sex-role categories in sample.

	Sex-role classification			
Gender	Masculine	Feminine	Androgynous	Undifferentiated
Males	29	17	34	23
	(28.2%)	(16.5%)	(33.0%)	(22.3%)
Females	23	33	36	32
	(18.5%)	(26.6%)	(29.0%)	(25.8%)

### Cattell Culture Fair IQ Distribution

IQ scores for the sample were normally distributed (Shapiro–Wilks *p* > 0.001) with a mean of 111.19 (*SD* = 14.21). As might be expected from a university subject pool, a one-sample *t*-test showed our sample mean was significantly higher than that of the general population, *t*(223) = 11.83, *p* < 0.001, *d* = 1.57. Additionally, an independent samples *t*-test confirmed that males and females in our sample did not differ significantly in measured intelligence, *t*(226) = 1.27, *p* = 0.206. Any observed gender difference in SEI could not, therefore, be explained by apparent differences in actual intelligence between groups resulting from sampling error. Additionally, a 2 × (Gender) 4 × (Sex-Role Category) factorial ANOVA confirmed no sex-role differences in measured intelligence, nor any interaction, all *F*s < 2.61, *p* > 0.05.

### Self-Estimated IQ Distribution

**FIGURE 2:**
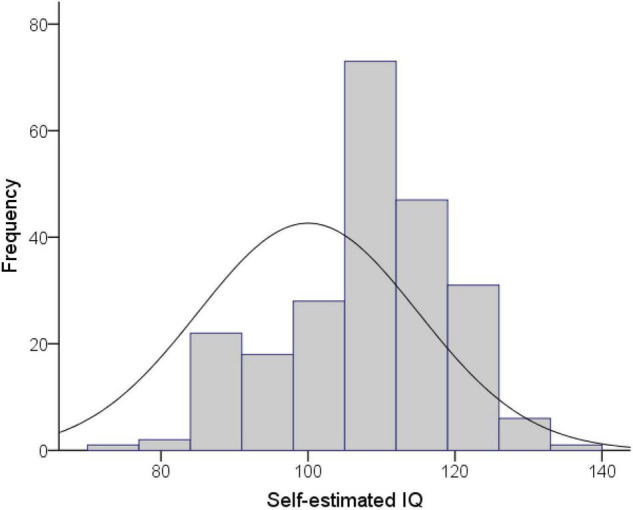
Histogram showing the distribution of self-estimated intelligence in the present sample, alongside the normal distribution of IQ scores in the general population. Our sample was significantly negatively skewed with the bulk of scores shifted to the right of the normal curve.

The distribution of self-estimated intelligence scores in our sample was significantly negatively skewed (std. skewness = −2.19, *p* = *0.028*), with a general tendency for participants to rate their intelligence as “above average,” and a mean SEI of 107.55 (*SD* = 10.98). Figure 2 presents a histogram of this distribution overlaid with the normal distribution of actual IQ scores in the general population (*M* = 100, *SD* = 15). Surprisingly though, approximately 19% of participants rated their intelligence as below average. This was somewhat unexpected as the “above average” effect had generally been regarded as robust—an issue we address further in the discussion. Additionally, there was a disproportionate number of females than males in this group, χ^2^ = 24.08, *p* < 0.001, with five males and 38 females rating their intelligence as below-average.

**FIGURE 3:**
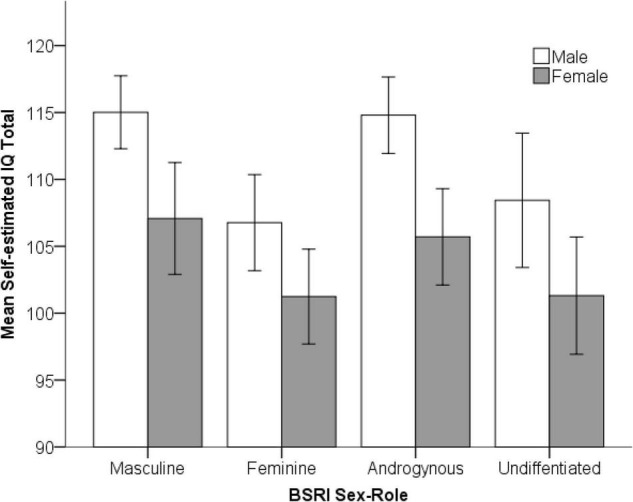
Self-estimated IQ scores across sex-role categories, for males and females (error bars represent the standard error of the mean).

A 2 × (Gender) 4 × (Sex-Role Category) factorial ANOVA^1^ was conducted on self-estimated IQ scores (see Figure 3). Although mild negative skewness was present (absolute standardized skewness = 2.23, *p* < 0.05), the ANOVA is robust against minor violations of normality when variances are equal (Field and Wilcox, 2017). The assumption of homogeneity of variance was met. As predicted by H1 there was a significant main effect of gender, *F*(1, 219) = 30.79, *p* < 0.001, η*^2^* = *0.12*. Males (*M* = 112.12, *SD* = 9.20) reported significantly higher self-estimated IQ than females (*M* = 103.66, *SD* = 10.88), *t*(225) = 5.55, *p* < 0.001, *d* = 0.74, which equates to a difference of approximately 8.5 IQ points. There was also a significant main effect of sex-role category, *F*(3, 219) = 7.23, *p* < 0.001, η*^2^* = *0.09*. A planned linear contrast compared the high masculinity participants (masculine + androgynous) to the low masculinity participants (feminine + undifferentiated). Consistent with H2, masculine and androgynous subjects gave higher self-estimates of IQ than feminine and undifferentiated, *t*(225) = 4.65, *p* < 0.001, *d* = 0.62. Both effects were medium in size. There was no significant interaction between gender and sex-role category.

### General and Academic Self-Esteem

**FIGURE 4:**
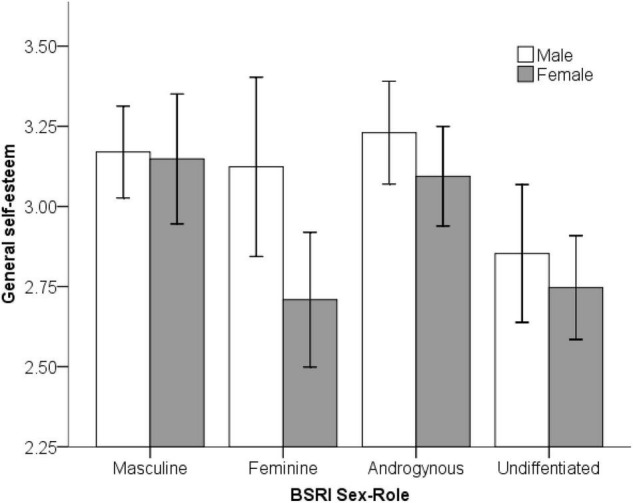
Rosenberg General Self-Esteem scores across gender and sex-role categories (error bars represent the standard error of the mean).

Next, we investigated individual differences in general and academic self-esteem, as these may make a contribution to perceptions of how intelligent our subjects perceived themselves to be. For the Rosenberg General Self-Esteem measure we conducted a 2 × (Gender) 4 × (Sex-Role Category) factorial ANOVA (see Figure 4). The data was normally distributed, and all assumptions were met. There was a significant main effect of gender, *F*(1, 219) = 6.71, *p* = 0.010, η*^2^* = 0.03, with males giving higher self-reports of general self-esteem than females (*d* = 0.40). Additionally there was a significant main effect of sex-role category, *F*(3, 219) = 7.88, *p* < 0.001, η*^2^* = 0.10, but no interaction between these terms. The effect of sex-role category was stronger than the social category of gender. In line with experimental hypotheses, a planned contrast confirmed that masculine and androgynous subjects reported higher general self-esteem scores than feminine and undifferentiated, *t*(225) = 4.62, *p* < 0.001, *d* = 0.62, which was a medium sized effect. Significant gender and sex-role differences indicate support for H3.

We repeated the factorial ANOVA for the academic self-esteem measure. As was the case with general self-esteem, males reported significantly higher academic self-esteem than females, *F*(1, 219) = 15.01, *p* < 0.001, η*^2^* = 0.06. A significant main effect of sex-role category, *F*(3, 219) = 6.04, *p* = 0.001, η*^2^* = 0.08, was also found. However, the interaction was not significant, and again the sex-role identification effect was slightly stronger than gender. The planned contrast demonstrated that participants with high masculinity (masculine and androgynous sex-roles) reported significantly higher academic self-esteem than participants with low masculinity (feminine and undifferentiated sex roles), *t*(225) = 4.26, *p* < 0.001, *d* = 0.57, which is a medium effect size.

### Bivariate Correlations

Bivariate correlations between all measures are reported in Table 2. Directions of correlations were consistent with previous literature, with gender and masculinity being significantly correlated with self-estimated IQ, both measures of self-esteem, and with IQ discrepancy scores (defined as self-estimated IQ—Cattel IQ). Additionally, self-estimated IQ was positively correlated with Cattell IQ scores, general self-esteem and academic self-esteem.

**TABLE 2:** Bivariate correlations between gender and sex-role measures, self-estimated intelligence, measured intelligence, general and academic self-esteem (*N* = 228).

Measure	1.	2.	3.	4.	5.	6.	7.	8.
1. Gender^a^	–	−0.21**	0.16*	−0.38***	–0.08	−0.20**	−0.20**	−0.27***
2. BSRI masculinity		–	0.02	0.34***	0.06	0.19**	0.37***	0.26***
3. BSRI femininity			–	–0.04	–0.07	0.04	0.11	–0.04
4. Self-estimated IQ				–	0.30***	0.44***	0.28***	0.45***
5. Cattell IQ					–	−0.72***	–0.02	0.08
6. IQ Discrepancy						–	0.22**	0.25***
7. Rosenberg Self-Esteem							–	0.54***
8. Academic Self-Esteem								–

*p < 0.05, **p < 0.01, and ***p < 0.001.

*^a^Dummy coded variable; 0 = male, 1 = female.*

### Predictors of Gender Differences in Self-Estimated Intelligence

Next, we set out to explore possible explanations for the male hubris, female humility effect. In the sample, the correlation between SEI and measured intelligence was just at the cusp of being medium in strength, *r*(228) = 0.30, *p* < 0.001, and the scatterplot confirmed it was linear in nature. This is consistent with past research that finds people are generally sound judges of their intelligence.

**FIGURE 5:**
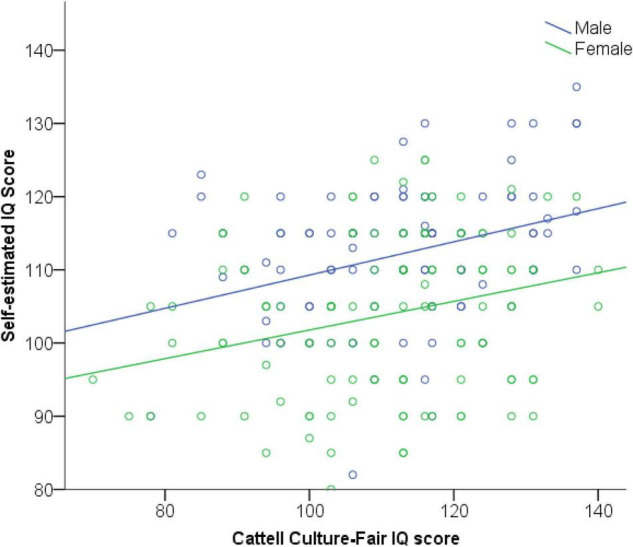
Scatterplot of association between self-estimated and psychometric IQ, for males and females, respectively.

One possible explanation for the MHFH effect might be that males and females greatly differ in the *accuracy* of their judgments of self-estimated intelligence though. To rule out this explanation, we examined the bivariate correlation between SEI and measured intelligence for males and females separately (see Figure 5). The correlation between SEI and measured intelligence was slightly higher for males, *r*(103) = 0.33, *p* < 0.001, than for females, *r*(124) = 0.26, *p* = 0.004, but again, both fell in the small to medium range of effect sizes and any difference most likely reflects sampling error. To confirm this, Fisher’s *r*-to*-z* transformation was applied to assess the significance of the difference between the two correlation coefficients *r*_male_ and *r*_female_, *z*_dif_ = 0.57, *p* = 0.284 (1-tailed), indicating no difference. Thus, we were able to rule out the possibility of differences in accuracy between males and females as an explanation for the male hubris, female humility problem. As can be seen in Figure 5 though, visual inspection does suggest a tendency for gender differences in direction, with more blue scores above the regression line and more green scores below.

Another plausible explanation for gender differences in SEI might be the contribution of self-esteem. Reported in Table 2, there was a moderate positive correlation between self-estimated intelligence and general self-esteem scores. However, it is also plausible that having a high intellect also makes a positive contribution to one’s general self-esteem, so we tested whether the correlation between SEI and general self-esteem remained significant after controlling for psychometric IQ. The positive correlation between SEI and Rosenberg General Self Esteem with CCFIT scores partialed out was still statistically significant, *r* = 0.30, *p* < 0.001, and of moderate strength (i.e., general self-esteem was associated with self-estimates of intelligence). As might be expected, the correlation between SEI and academic self-esteem was somewhat stronger, *r* = 0.45, though this is likely to be a bidirectional relationship.

To explore the joint effects of the social category of gender, sex-role identification, and general self-esteem, a hierarchical multiple regression was conducted on self-estimated intelligence scores (see Table 3). Psychometric IQ scores were entered at Step 1 in order to control for individual differences in actual intelligence, *F*_chg_(1,223) = 22.71, *p* < 0.001, explaining approximately 9% of the variance in SEI. Next in Step 2, gender was entered in conjunction with sex-role identification (BSRI masculinity and femininity scores). Although only gender and masculinity were hypothesized to make a significant contribution to SEI scores, femininity was included to consider the possibility it also made a significant contribution. Together these factors resulted in an increased model fit, *F*_chg_(3,220) = 20.76, *p* < 0.001, explaining an additional 20% of variance in the dependent variable of SEI. Both gender and masculinity scores were significant predictors. Finally at Step 3, General Self-Esteem scores were entered to test the hypothesis that self-esteem may still be a contributing factor. This resulted in a small increase in model fit, *F*_chg_(1,219) = 4.39, *p* < 0.001. The final model was statistically significant, *F*(5, 219) = 19.36, *p* < 0.001, accounting for 31.7% of the variance in individual self-estimates of intelligence. As can be seen from the table, even after controlling for individual differences in measured intelligence (β = 0.27), the three hypothesized predictors of gender, masculinity and general self-esteem made significant and unique contributions. Gender was the strongest predictor, followed by measured intelligence, masculinity, and finally a smaller contribution of general self-esteem which had considerable overlap with the other predictors.

**TABLE 3:** Hierarchical multiple regression of self-estimated intelligence scores (*N* = 228).

Variable		β	*t*	*p-value*	*sr* ^2^	*R*	*R* ^2^
Step 1						30	0.09
	Cattell IQ	0.30	4.70	<0.001***	0.09		
Step 2						0.54	0.29
	Cattell IQ	0.26	4.57	<0.001***	0.06		
	Gender (0 = male)	–0.31	–5.33	<0.001***	0.10		
	Masculinity	0.28	4.89	<0.001***	0.06		
	Femininity	0.02	0.39	0.700	0.00		
Step 3						0.55	0.31
	Cattell IQ	0.26	4.72	<0.001***	0.07		
	Gender (0 = male)	–0.29	–4.95	<0.001***	0.08		
	Masculinity	0.23	3.80	0.001**	0.05		
	Femininity	0.01	0.11	0.913	0.00		
	General Self-Esteem	0.13	2.19	0.030*	0.02		

*p < 0.05, **p < 0.01, and ***p < 0.001.

### Statistical Mediation

**FIGURE 6:**
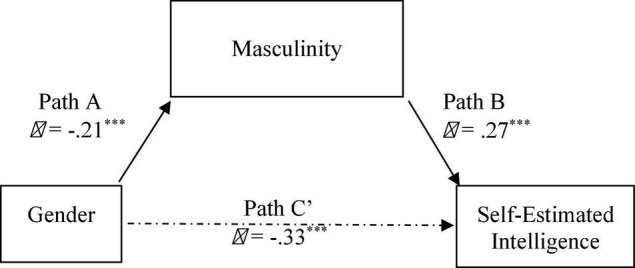
Indirect effect of gender on SEI, with masculine sex-roles acting as a mediator on self-estimated intelligence. Path C’ represents the direct effect of gender after controlling for the mediator. *** *p* < 0.001.

We next examined whether masculine sex-role identification (masculinity score as a continuous variable) acted as a statistical mediatior in the relationship between gender and SEI scores. Baron and Kenny (1986) proposed three criteria for establishing statistical mediation. Firstly, the predictor (gender) should predict the dependent variable (SEI). Secondly, the predictor must be correlated with the proposed mediator variable (masculinity, shown as Path A). Thirdly the mediator must correlate with the dependent variable (SEI) even after controlling for the contribution of the predictor (shown as Path B). The Sobel test of statistical mediation was significant, Sobel *z* = −2.55, *p* = 0.010, and calculation of the bootstrapped estimate of the indirect effect showed that it differed significantly from zero (95% CI = −2.26 to −0.41), following the bootstrapping criteria outlined in Preacher and Hayes (2004). As the mediation effect was significant, we then tested whether the relationship was fully or only partially mediated (Baron and Kenny, 1986). In a full mediation model, the association between predictor and dependent variable will no longer be statistically significant after controlling for the mediator (i.e., all of the effect of the predictor acts indirectly through the mediator, and does not make a direct contribution). This relationship is represented by Path C in Figure 6. Though diminished, the beta weight remained statistically significant, indicating that the relationship was only a partial mediation. Though acting indirectly through masculine sex-role identification, there was still a direct contribution of gender to SEI scores.

Having identified in the multiple regression analysis that biological sex made a slightly stronger contribution to SEI than measured intelligence, sex-role identification, and general self-esteem, we sought to quantify how large the discrepancy between self-estimates and measured intelligence was. A composite variable representing the difference between self-estimated and measured intelligence was created, with positive values indicating higher SEI than measured intelligence. An independent samples *t*-test on IQ discrepancy scores confirmed a significant gender difference, *t*(225) = 3.04, *p* = 0.003, *d* = 0.40. Visual inspection of the discrepancy scores showed that *on average*, males in our sample demonstrated fairly sound judgment in appraising their intelligence (*M* = −0.35, *SD* = 13.61), but that there was also wide variability with some males greatly overestimating their intelligence and some males underestimating (range = −27 to + 38 IQ points). However, females systematically *undervalued* their intellectual capabilities by over six IQ points (*M* = −6.34, *SD* = 15.83), and for those female participants that did offer inflated self-estimates, these were much smaller in size (range = −41 to + 25 IQ points). Only the female discrepancy scores differed significantly from zero however (*p* < 0.001).

Next, a 2 × (Gender) 4 × (Sex-Role Category) factorial MANOVA was performed on the nine self-estimates of Gardner’s multiple intelligences. As the cell size differed across sex-role category and Box’s *M* was significant (*p* < 0.001), Pillai’s trace was selected as the more conservative estimate. Assumptions of normality and homogeneity of variance were met. In line with previous research, there was a significant multivariate effect of biological sex, *F*(9, 212) = 7.02, *p* < 0.001, η*^2^* = *0.23*, which is a medium to large effect. There was also a significant multivariate effect of sex-role identification, *F*(27, 642) = 2.22, *p* < 0.001, η*^2^* = *0.09*, though there was no significant interaction, *F*(27, 642) = 1.02, *p* = 0.437. As the overall multivariate effects were significant and of non-trivial size, this justified examination of univariate effects without a need to apply a Bonferroni correction (c.f., Huberty and Morris, 1989). For ease of comparison, sex and sex-role differences are reported separately in Tables 4, 5, respectively. Five of the nine multiple intelligence domains showed significant differences between males and females, with effect sizes ranging from small to large.

**TABLE 4:** Gender differences on self-estimated multiple intelligences.

Domain	Male	Female	*F*_(1,220)_	*p*-value	*d*
1. Verbal	106.45 (12.87)	107.07 (11.65)	0.73	0.395	–0.05
2. Logical-Mathematical	108.39 (16.68)	98.66 (13.43)	18.36	< 0.001***	0.64
3. Spatial	109.80 (12.51)	98.54 (11.93)	40.79	< 0.001***	0.92
4. Musical	102.64 (18.11)	99.50 (14.72)	0.64	0.426	0.19
5. Bodily-kinesthetic	112.57 (14.26)	106.47 (14.74)	7.54	0.007**	0.42
6. Interpersonal	112.69 (12.98)	112.86 (11.72)	0.20	0.654	–0.01
7. Intrapersonal	110.61 (12.63)	109.36 (12.79)	0.11	0.742	0.09
8. Naturalistic	104.43 (11.88)	99.10 (11.06)	10.36	0.001**	0.46
9. Existential/spiritual	108.72 (16.92)	102.94 (12.84)	6.85	0.009**	0.39

*p < 0.05; **p < 0.01; ***p < 0.001.

**TABLE 5:** Sex-role differences in self-estimated multiple intelligences.

Domain	Masc.	Fem.	Andr.	Undif.	F-ratio	Planned Contrast
1. Verbal	110.35 (1.61)	104.51 (1.81)	106.74 (1.51)	104.54 (1.66)	2.78*	*t*(226) = 2.44, *p* = 0.015, *d* = 0.33
2. Logical-Mathematical	106.66 (1.98)	98.41 (2.22)	104.38 (1.85)	103.23 (2.04)	2.68*	*t*(226) = 2.32, *p* = 0.021, *d* = 0.32
3. Spatial	105.71 (1.59)	100.34 (1.79)	107.01 (1.49)	101.83 (1.64)	3.70*	*t*(226) = 3.22, *p* = 0.001, *d* = 0.43
4. Musical	102.98 (2.12)	95.86 (2.38)	105.23 (1.99)	97.40 (2.18)	4.25**	*t*(226) = 3.44, *p* = 0.001, *d* = 0.46
5. Bodily-kinesthetic	112.18 (1.86)	106.22 (2.10)	114.00 (1.75)	103.96 (1.92)	6.47***	*t*(226) = 4.18, *p* < 0.001, *d* = 0.56
6. Interpersonal	113.82 (1.53)	112.36 (1.72)	117.87 (1.44)	105.83 (1.58)	10.82***	*t*(226) = 4.30, *p* < 0.001, *d* = 0.57
7. Intrapersonal	110.48 (1.66)	107.58 (1.87)	113.88 (1.56)	106.44 (1.71)	4.09**	*t*(226) = 3.03, *p* = 0.003, *d* = 0.40
8. Naturalistic	101.83 (1.50)	100.49 (1.69)	104.20 (1.41)	99.12 (1.55)	2.15*	*t*(226) = 2.09, *p* = 0.038, *d* = 0.27
9. Existential/spiritual	106.80 (1.93)	104.23 (2.17)	110.47 (1.81)	100.57 (1.99)	4.79**	*t*(226) = 3.15, *p* = 0.002, *d* = 0.42

*p < 0.05; **p < 0.01; ***p < 0.001.

Table 5 presents sex-role differences across the nine multiple intelligence domains. Although gender differences were not present for every domain (Table 4), there were significant sex-role differences for each of the domains. Accordingly, a planned linear contrast was conducted comparing the high masculinity groups (masculine + androgynous) with the low masculinity groups. Masculine persons reported significantly higher self-estimates of multiple intelligences, with effect sizes ranging from quite small to medium in size.

## Discussion

The goal of the present study was to investigate psychological factors that contribute to the widely observed male hubris, female humility effect. These include baseline psychometric intelligence, general self-esteem, and sex-role identification (masculine and feminine personality traits). By including a suitable measure of psychometric intelligence, we were also able to rule out certain explanations for the MHFH effect, such as genuine differences in measured intelligence between the males and females recruited due to sampling bias, as well as discrepancies in the accuracy of self-estimated intelligence in one or both genders. What we found was a more nuanced picture that gives support to a multifactorial model for explaining gender differences in self-estimated intelligence. Furthermore, we found sex and sex-role differences for some but not all multiple intelligence domains, consistent with cultural gender stereotypes about certain intellectual domains. We first review support for the experimental hypotheses and then discuss the important social and educational implications of this pattern of results.

Hypothesis 1 was supported, with males reporting higher SEI scores than females as found in previous studies (Syzmanowicz and Furnham, 2011). Our observed effect (*d* = 0.74) was somewhat larger than that typically reported but in line with some studies reporting quite large gender differences (Bennett, 1996; Zhang and Gong, 2001). It may be driven in part by the sex-role composition of our sample as the planned contrast confirmed a similarly large sex-role effect (*d* = 0.62) between high and low masculinity subjects which supported Hypothesis 2. A previous study by Szymanowicz and Furnham (2013) also found sex-role differences, with masculinity predicting self-estimates of general intelligence and multiple intelligences consistent with our study.

However, inspection of the distribution of SEI scores highlights some key differences to that typically described in SEI studies. The better-than-average effect is widely regarded as a truism in the literature (Alicke et al., 1995) for it has been so widely reported (for a review see Kruger and Dunning, 1999). Paulhus et al. (1998) claim that “rarely do people rate themselves as “below average,” p. 526. It is difficult to determine with any certainty how valid such claims are, however, as despite the large number of studies on self-estimated intelligence histograms are rarely presented. But on inspecting the histogram in Figure 2, there were an inordinately high number of participants that saw themselves as having below average intelligence. The stimulus material provided an anchoring frame of 100 as “average intelligence,” following the methodology of Furnham and colleagues. So it is unclear whether this results from a fundamental difference in the composition of our observed sample, or is instead a Hawthorne effect from the knowledge that subjects would soon complete a psychometrically valid IQ test. As noted, a number of studies use a proxy test like the Raven’s Progressive Matrices, or the Wonderlic Personnel Test, but these stimulus materials do not explicitly identify them as IQ tests; thus they may be less imbued with power in the minds of our participants as an “actual IQ test.” Might this have tempered somewhat the tendency to give inflated self-estimates for social desirability reasons, and altered behavior accordingly? Alternately, differences in the characteristics of our sample may be responsible, given the percentage of students from non-psychology faculties and broader diversity of sex-roles. Only replications of the study will be able to shed more light on this matter, but we strongly recommend that future studies report information on the distribution of SEI scores and the number of subjects who rate themselves as below average. It is also possible that it may be tapping into personality traits such as honesty-humility, as found by previous studies (Kajonius, 2014).

Consistent with previous studies, we also found predicted gender differences in general self-esteem and of comparable size (Gentile et al., 2009). Additionally, masculine/agentic personality traits appear to confer benefits for overall self-esteem as well as academic self-esteem (Whitley, 1983; Hirschy and Morris, 2002), which is important as this hypothesis has rarely been examined in recent years and may have been subject to shift as gender-norms change. Observed correlations between masculinity and general self-esteem (*r* = 0.26) are of similar strength to those reported in other studies (Hirschy and Morris, 2002). Thus, both sex and sex-role identification contribute jointly to self-esteem, affirming Hypothesis 3. To our knowledge, few researchers have empirically tested the contribution of general self-esteem to self-estimates of general intelligence. Only a single study, by Mirjalili et al. (2011) could be located, finding a correlation between Rosenberg Self-Esteem scores and SEI of *r* = *0.32* in a sample of Iranian boys and girls. Gender differences in general self-esteem though may in turn contribute to SEI scores, though causation cannot be established with a correlational design.

Subjects in our study were also reasonably astute judges of their own intellectuality, with a moderately sized positive correlation between SEI and psychometric IQ, consistent with Hypothesis 4. The observed effect size was comparable to previous studies (Paulhus et al., 1998), and one possibility that we were able to rule out as an explanation for the MHFH was that one or both genders held “completely unrealistic” views of their abilities as might be suggested from the label of male hubris or female humility. Instead, what emerged from the results of the multiple regression analysis was a multifactorial explanation for the MHFH effect. Affirming Hypothesis 5, there were significant independent contributions of biological sex, masculinity, and general self-esteem on self-estimates even after controlling for the contribution of psychometrically measured intelligence. Furthermore, the association between biological sex and SEI was statistically mediated by masculinity.

Additional investigation of discrepancy scores showed that males were fairly close in their self-estimates (but keep in mind this may have been tempered by the knowledge their estimates would be compared against their pending IQ test scores). However, there was still a tendency in females to underestimate their intelligence by an average of 6.32 IQ points (or almost half a standard deviation). This, when combined with the self-esteem and sex-role mediation effects, offers a broader explanation for the MHFH effect—we have a more nuanced picture than rather than simply the social category of male and female determining self-estimates.

While there was firm evidence for gender and sex-role differences in self-estimates of “global” intellectual ability for our sample, this did not extend to all multiple intelligences (Hypothesis 7). Consistent with Syzmanowicz and Furnham’s (2011) meta-analysis there were gender differences for logical-mathematical as well as spatial intelligence which are stereotypically regarded as masculine and which the authors termed domain-masculine IQ (DMIQ). Across the stereotypically feminine intelligence domains of verbal, interpersonal and intrapersonal (collectively regarded by laypersons as “emotional intelligence”) we did not see evidence of male hubris. However, despite being relatively small in magnitude, we did observe significantly higher male estimates for bodily-kinesthetic, naturalistic and existential/spiritual intelligence. In addition, there was firm support for sex-role differences in multiple intelligences, with masculine and androgynous subjects reporting higher estimates than feminine and undifferentiated. This also replicates results found in a previous study by Szymanowicz and Furnham (2013). Somewhat surprisingly however, these effects were observed even on stereotypically feminine intellectual domains, though again, the effect sizes were small-to-medium. The previous study failed to find significant sex-role differences on emotional intelligence (represented by inter- and intra-personal intelligence in the current study).

### Social and Educational Implications

Though widely observed, for decades researchers have struggled to identify and understand the psychological factors contributing to the male hubris, female humility effect. While a mild self-enhancing bias may be protective and to some degree self-fulfilling, the psychological consequences of inaccurately calibrated estimates of intellectual ability can also be damaging. Unrealistically inflated estimates may set students up for future discouragement and failure if their reach exceeds their grasp: in the United States, college completion rates have been steadily declining and disproportionately affect males (Bound et al., 2010), while the percentage of Ph.D. students who start but do not complete a Ph.D. exceeds 50% (Most, 2008). Perhaps even more problematic though is the effect of underestimation on achievement motivation, course selection and educational aspirations (Eccles, 2013): if you tell yourself that you can’t, then you’re right—you won’t. Systemic differences in self-estimated intelligence for an entire social class (women), has serious social, educational and financial implications that cannot be understated. Furthermore, we found evidence that self-esteem and sex-role identification are also predictors of female hubris—and one must be mindful that there were also males in our sample who saw themselves as below average. Educators should also be reminded of the effect that praise and encouragement can have for students who underestimate their abilities, as well as the effect that negative gender stereotypes and implicit bias can have on impressionable young students. While disingenuous or inflated praise can sometimes backfire (“the praise paradox,” Brummelman et al., 2014), as educators we should also be mindful that students might not always recognize their full potential. Dweck (2002) notes that strategic praise can increase academic motivation in such students (particularly when paired with a growth mindset), which also has a follow-on effect with course selection (Eccles, 2013).

Parental beliefs about differential intellectuality of sons and daughters reflect larger cultural beliefs that implicitly associate men and masculinity with intelligence (Nosek et al., 2002). Research on intelligence is unequivocal that men and women do not differ in objectively measured intelligence (Neisser et al., 1996), and endorsement of explicit beliefs that one gender is superior is quite rare (Swim, 1994). However, implicit beliefs differ, as do parental estimates of the intelligence of sons and daughters (Beloff, 1992; Furnham and Rawles, 1995). Rigid adherence to gender-roles, particularly in the educational context is problematic. Socio-cultural transmission of gender stereotypes (through parents, teachers, peers and media) may be in part fueling this phenomenon (Kollmayer et al., 2018), but there’s also individual variability in the extent to which these are internalized. Masculine personality traits appear to be a protective factor, as well as predicting general self-esteem.

### Limitations and Directions for Future Research

A previous study by Kajonius (2014) found that the personality trait of honesty-humility also predicted self-estimated intelligence, and that there are likely social desirability factors at play. Additionally, it is plausible that some participants may have had knowledge of either the male-hubris/female humility effect, or that of the Dunning-Krueger effect. There is also the possibility that students’ self-perceptions of intellectual ability were shaped by being in an environment where they are provided feedback on assignments and examinations. While the ethnicity of students was not recorded, having an ethnically diverse sample which included international students might introduce the possibility of stereotype threat effects (Steele and Aronson, 1995), where students internalize negative stereotypes about particular ethnic groups.

As acknowledged above, we are also uncertain whether the number of people self-estimating themselves as below-average intelligence is a Hawthorne effect due to their knowledge that we would measure psychometric intelligence, or instead a difference in the composition of our sample. This is a point of difference from most previous studies on self-estimation of intelligence, as most previous studies have not coadministered an intelligence test. By their nature, university students are a self-selected sample and in Australia, go through the bottleneck of meeting certain educational achievement requirements. Could it be that lowered admission requirements and alternate entry pathways resulted in a markedly different sample to that found in typical American college subject pools? Or might the below-average effect observed here have been overlooked in previous studies? Further research is needed to explore this issue, and we advocate for coadministering measures of self-esteem and sex-role identification. It is hoped that further research will elucidate whether it is biological sex or psychological gender that better explains gendered patterns of self-estimated intelligence.

## Conclusion

Possible explantions for the widely observed gender differences in self-estimated intelligence were investigated, which has also been termed the male-hubris female humility effect. We found that the issue is complex and nuanced, with no single cause emerging but rather that there were a number of contributing factors. Firstly, sex-role identification makes a significant contribution to intellectual self-image, with masculine/agentic personality traits leading to higher self-estimates. Secondly, we found a significant and independent effect of self-esteem to self-estimated intelligence. As females in our sample reported lower general self-esteem in line with the trend identified in the literature, this may be a strong factor underlying the male-hubris female humility effect. However further research is needed to elucidate the risk factors that identify patterns of over-/under-estimation of intelligence.

## Data Availability Statement

The raw data supporting the conclusion of this article will be made available by the authors, without undue reservation.

## Ethics Statement

The studies involving human participants were reviewed and approved by Human Research Ethics Committee, Griffith University. The patients/participants provided their online informed consent to participate in this study.

## Author Contributions

DR, DN, and GA contributed to the conception and design of the study and contributed equally to the interpretation. DR completed the recruitment of participants and data analysis. All authors contributed to manuscript revision, read and approved the submitted version.

## Conflict of Interest

The authors declare that the research was conducted in the absence of any commercial or financial relationships that could be construed as a potential conflict of interest.

## Publisher’s Note

All claims expressed in this article are solely those of the authors and do not necessarily represent those of their affiliated organizations, or those of the publisher, the editors and the reviewers. Any product that may be evaluated in this article, or claim that may be made by its manufacturer, is not guaranteed or endorsed by the publisher.

## Footnotes

1 A reflected log transformation was applied to the distribution and the analysis repeated, with no change in outcome. As the untransformed data was in a metric (IQ score) that was more meaningful, the untransformed data is reported. Additionally, the analysis was run with CCFIT as a covariate with no change in outcome.
